# *In Situ* AFM Imaging of Microstructural Changes Associated with The Spin Transition in [Fe(Htrz)_2_(Trz)](Bf_4_) Nanoparticles

**DOI:** 10.3390/ma9070537

**Published:** 2016-06-30

**Authors:** María D. Manrique-Juárez, Iurii Suleimanov, Edna M. Hernández, Lionel Salmon, Gábor Molnár, Azzedine Bousseksou

**Affiliations:** 1 LCC, CNRS & Université de Toulouse (UPS, INP), Toulouse 31077, France; dolores.manrique@lcc-toulouse.fr (M.D.M.-J.); Iurii.Suleimanov@lcc-toulouse.fr (I.S.); lionel.salmon@lcc-toulouse.fr (L.S.); 2 LAAS, CNRS & Université de Toulouse (INSA, UPS), Toulouse 31077, France; 3 Department of Chemistry, National Taras Shevchenko University, Kiev 01601, Ukraine; 4 Facultad de Ciencias, Universidad Nacional Autónoma de México, Mexico City 04510, Mexico; ednah@ciencias.unam.mx

**Keywords:** spin crossover, microstructures, atomic force microscopy

## Abstract

Topographic images of [Fe(Htrz)_2_(trz)](BF_4_) nanoparticles were acquired across the first-order spin transition using variable-temperature atomic force microscopy (AFM) in amplitude modulation mode. These studies revealed a complex morphology of the particles consisting of aggregates of small nanocrystals, which expand, separate and re-aggregate due to the mechanical stress during the spin-state switching events. Both reversible (prompt or slow recovery) and irreversible effects (fatigue) on the particle morphology were evidenced and correlated with the spin crossover properties.

## 1. Introduction

The change of the molecular and crystal structure in spin crossover (SCO) complexes has been extensively investigated in the past for its central role in the SCO mechanism [1,2,3]. Based on extensive X-ray crystallographic studies (for examples see References [3,4,5,6,7]), today it is well established that the population of anti-bonding orbitals in the high spin state of the complex leads to a ubiquitous increase of metal-ligand bond lengths and of the volume of the coordination polyhedra. For example, in the case of the most common Fe^II^N_6_ coordination sphere, a ~10% (average) increase of the Fe-N bond lengths and a ~25% increase of the octahedron volume were systematically observed when going from the low spin (LS) to the high spin (HS) state. This change of metal-ligand bond lengths implies a drastic change of the ligand field strength; hence, it can be considered as the driving force of the SCO at the molecular level [8]. In addition, the breathing of the metal coordination sphere further propagates at the intra- and inter-molecular levels in SCO solids. This leads to sizeable elastic interactions between the molecules, manifested by first-order spin transitions, hysteresis, self-acceleration and other collective properties. These phenomena are well understood today [9], even if quantitative structure-property relationships remain extremely difficult to establish due to the structural complexity and diversity of these materials as well as the rather small energy differences, which should be calculated.

Beyond the molecular and crystal structure, the spin transition is also expected to be altered by the microstructure of the material as a result of the strong coupling between the electronic and elastic degrees of freedom. Microstructures can be defined as micro- and nanometer-scale inhomogeneities, which include dislocations, microtwins, domains structures, grain boundaries, etc. It is well known that microstructures can strongly alter the macroscopic properties of materials and they often play an important role in phase transitions. In the case of the SCO phenomenon, microstructural effects have been inferred through sample grinding experiments, which usually led to the narrowing of the hysteresis as well as more gradual and less complete spin transitions (see Reference [4] and the references therein). In addition, significant microstructural changes may also occur during the spin transition due to the non-negligible lattice misfit between the two phases. For example, a fragmentation of crystals upon SCO and associated property changes was reported in Reference [10]. Despite these observations, the microstructural aspects of the SCO phenomenon are often overlooked. In addition, the reported studies usually focus only on the correlation between sample morphologies and SCO properties and do not consider the possible role of reversible/irreversible microstructural changes in the course of the spin transition.

Recently, a detailed crystallographic study has been devoted to the investigation of both lattice and microstructural properties of nanocrystalline powders of the [Fe(Htrz)_2_(trz)](BF_4_) (Htrz = 1H-1,2,4-triazole, trz = 1,2,4-triazolato) SCO complex 1 [11,12]. This compound crystallizes in an orthorhombic (Pnma) structure with drastically different lattice parameters in the LS (a = 17.3474 Å, b = 7.3247 Å, c = 9.1907 Å) and HS (a = 17.4968 Å, b = 7.7874 Å, c = 9.5643 Å) states, corresponding to a volume expansion of ca. 11% associated with the SCO. The crystal structure consists of “infinite” [Fe(trz)(H-trz)_2_]*_n_*^*n*+^ chains linked together by the BF_4_^−^ counter-ions as well as hydrogen bonds [13]. The spin transition in 1 occurs with a broad hysteresis of ca. 30 ± 10 K centered around 360 ± 10 K, the exact values depending on the synthesis conditions. This compound has attracted much attention from the SCO community [14] for its broad bistability range centered above room temperature [15], the preservation of this bistability even in particles a few nm in size [16], and its high robustness. Indeed, after an initial “run-in” during the first heating cycle, samples of 1 exhibit an extremely well reproducible spin transition. The SCO in 1 was shown to persist over several thousand switching events in ambient air, though a slow and continuous decrease of the hysteresis width from 44 K to 27 K was also observed all the way through 3000 thermal cycles [17]. The crystallographic study of Grosjean et al. [11,12,13] was carried out on sub-micrometer-size particles, which were revealed to actually be aggregates of cylindrical nanocrystallites a few hundred nanometers in length. These particles were submitted to 50 thermal cycles and the evolution of their crystal structure and microstructure was followed in situ by means of powder X-ray diffraction (PXRD). It turned out that the unit cell, in particular parameter a, is slightly modified during the first few cycles, but remains well reproducible afterwards. It was thus suggested that the initial “run-in” of the sample occurs not due to solvent loss as it was previously suggested, but as a result of a small ordering of the lattice. This finding was also supported by the analysis of microstrains in the sample, which decreased after the first cycle (defect annealing) and remained constant over subsequent cycles. On the other hand, the authors pointed out a significant structural fatigability reflected by a continuous and highly anisotropic decrease of the coherent domain size along the crystallographic *b* direction (i.e., along the Fe-triazole chains) over successive thermal cycles. It is tempting to correlate this microstructural fatigue with the reported slow change of the hysteresis width, but this remains only a hypothesis at this stage.

In situ microscopy imaging techniques can also reveal important details about the particle morphology and microstructures as well as about their evolution during the spin transition. Up to now, mainly optical microscopy has been used for this aim in the SCO field (for examples see References [18,19] and references therein), but the limited spatial resolution of far-field optics makes this technique more suitable for the study of large single crystals several tenths of micrometers in size. In situ scanning probe microscopy [20,21,22,23,24] and electron microscopy [25] techniques have been only very recently employed on SCO materials, opening up exciting perspectives for the high spatial resolution analysis and manipulation of SCO objects in a broad size range. Here we present a variable-temperature AFM topography study of a nanocrystalline film of **1** with the aim to give a more comprehensive picture of the microstructural changes associated with the SCO in this sample.

## 2. Results and Discussion

**Figure 1:**
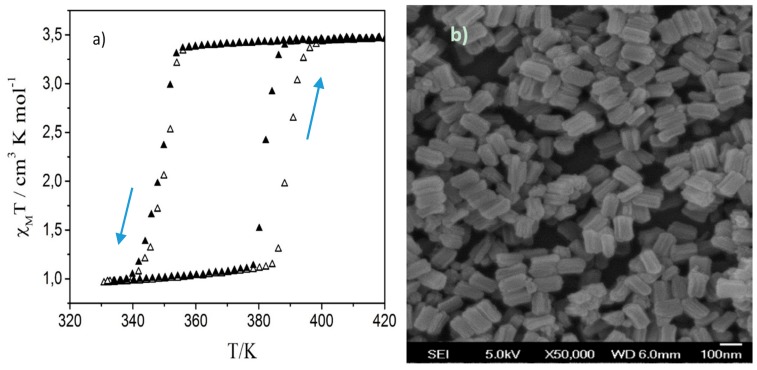
(**a**) Temperature dependence of the product of molar magnetic susceptibility and temperature for **1** over the first (open symbols) and second (closed symbols) thermal cycles. Heating and cooling are indicated by arrows; (**b**) Representative SEM image of the particles of 1.

Figure 1a presents the spin transition curve of **1** obtained from magnetic susceptibility measurements during the first two thermal cycles. The LS-to-HS transition occurs around 387 K (382 K) during the first (second) heating while the reverse transition occurs around 348 K on cooling in both cycles. SEM images of the as-synthesized sample (Figure 1b) reveal sub-micrometric platelet-like particles, which seem to be composed of several tightly aggregated rod-shaped crystallites, which are aligned along their axes. These images closely resemble those reported by Grosjean et al. [11,12]. Based on their PXRD data, these authors identified the rod-shaped crystallites as coherent domains of **1**. On this basis a parallel between the long axis of the particles and the b unit-cell parameter was also established.

**Figure 2:**
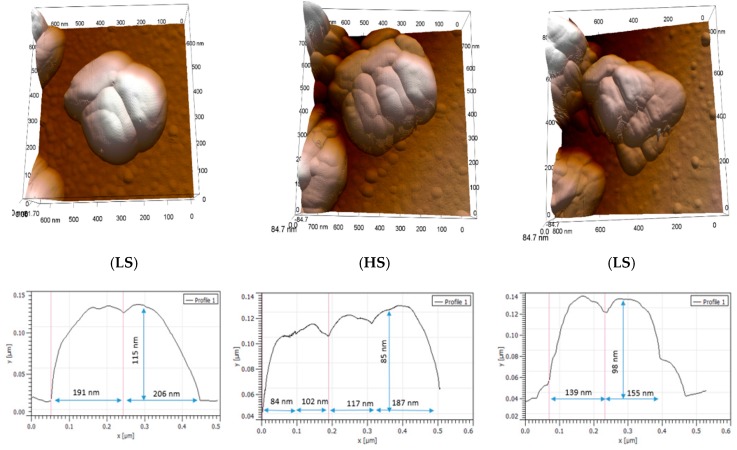
AFM height images and cross-sections of a particle of **1** acquired in different spin states at 358 K over a complete thermal cycle. From left to right: LS, HS and LS states. Images were recorded at 512 × 512 pixels at a line rate of 2 Hz. The z-scales range from 0 to 168 nm.

Figure 2 shows selected AFM topography images of a particle of 1 (see Figure S1 in the Supplementary Materials (SM) for further images from this experiment). Each image was acquired at the same temperature (358 K) within the hysteresis region successively in the LS state (as-received sample), then in the HS state after the first switching event, and finally in the LS state following a complete thermal cycle. One important point that can be extracted from this experiment is the apparent particle volume expansion which occurs in the HS state. This volume expansion reflects, however, not only the lattice expansion previously observed by PXRD, but also a rearrangement of the crystallites, which constitute the particle. In particular a significant change of the grain boundary morphology is obvious in these images. The crystallites are getting more separated in the HS state—presumably due to the mechanical stress, which arises during the spin transition. On the other hand it is difficult to analyze changes of individual crystallites. Following a complete thermal cycle, the initial morphology of the particle is not recovered, i.e., the spin transition leads to irreversible changes of the microstructure. Nevertheless, we observe a partial recovery of the initial particle shape through the stacking of the crystallites and a decrease of the particle size during the reverse HS-to-LS switch. Further thermal cycling gave rise also to morphological changes (see Figures S2 and S3 in the SM for examples), but these were always less substantial than those observed during the first cycle. At this point it may be worth underlining that we studied the topography changes on two different samples using different tips, different scan parameters and even two different AFM instruments over several complete thermal cycles and the main findings were very reproducible (see SM).

**Figure 3:**
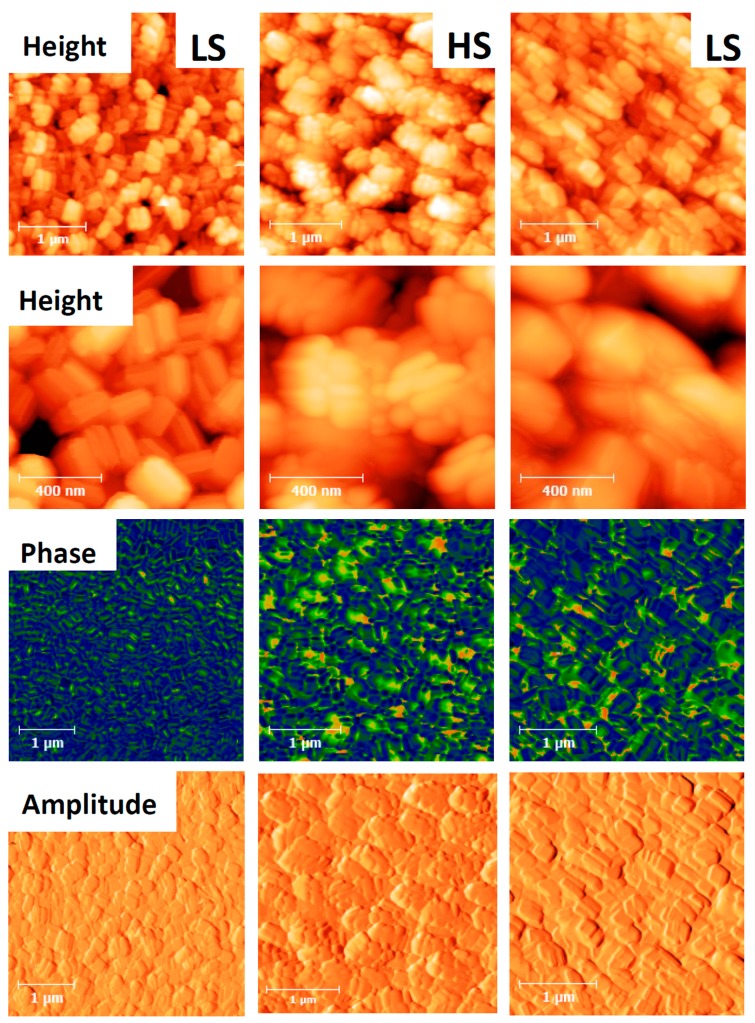
AFM images of **1** acquired during a complete thermal cycle. From left to right: LS (303 K), HS (360 K) and LS (360 K) states. From top to bottom: height (scan size: 3 × 3 μm^2^ and 1 × 1 μm^2^), phase and amplitude signals. Images were recorded at 512 × 512 pixels at a line rate of 1 Hz. The z-scale ranges from 0 to 300 nm (height) and from 0 to 90 degrees (phase).

A similar experiment is shown in Figure 3 for a dense film of particles of 1. In this case the sample was first thermally cycled three times between 298 and 393 K, left for 60 days in ambient air and then AFM images were acquired at 303 K and 360 K (in both spin states). The topography images show similar morphology changes to those discussed above for the previous experiment on the fresh sample, suggesting a slow recovery of the initial grain boundary morphology in ambient conditions (see also the images of the fresh sample in Figure S2). Notably, one can observe a substantial expansion of the particles in the HS state and at the same time the individual nanocrystals appear more separated. When going back to the LS state, the initial shape of the particles is partially recovered though the stacking of the crystallites, but the initial structure cannot be observed anymore. The topography changes are even more perceptible in the error (i.e., amplitude) signals. It is interesting to also compare the surface roughness (Table 1) in a quantitative manner through the parameters Ra and Rq, which are the arithmetic and root mean squared averages of the vertical deviations of the roughness profile, respectively. It is clear that for both Ra and Rq, roughness tends to decrease in the HS state, which may be explained by the separation of the nanocrystallites triggered by the spin-state switching. Overall our observations seem to be in agreement with the PXRD data of Grosjean et al. [11,12], where the coherent domain size was observed to exhibit an important size reduction during the first heating (at least 40%) with respect to the following ones and the LS domain size did not recover its original value. The change of the grain boundaries and particle orientations observed in our images are also in line with the mosaicity changes extracted from PXRD measurements. On the other hand, it is necessary to have in mind that under different preparation and storage conditions, these particles tend to (re)organize in a different manner. For this reason, morphology changes between the different samples may not be exactly the same.

Surprisingly, the quantitative analysis of the AFM phase signal did not reveal a significant change on the spin transition. In the HS state, the average phase angle shift is very similar to that in the LS state (14.4° vs. 14.9°) at the same temperature. This result is somewhat surprising as the surface stiffness of the particles in the HS phase is expected to be significantly lower, which should be reflected by the phase images [20]. However, the cross-talk between the topography changes and the phase signal may hide the effect related to the variation of the sample visocoelastic properties [26]. This issue will require further studies using quantitative nanomechanical techniques.

**Figure 4:**
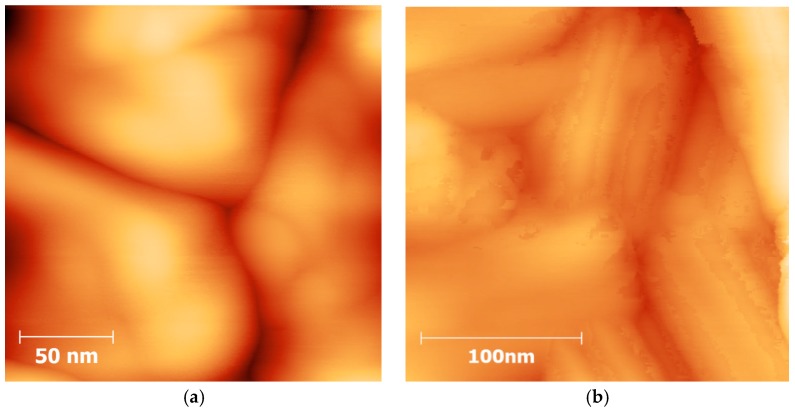
High resolution AFM topography images acquired at 303 K (**a**) on the fresh sample and (**b**) after eight thermal cycles. Images were recorded at 512 × 512 pixels at a line rate of 2 Hz. The scan size was (**a**) 200 × 200 nm^2^ and (**b**) 220 × 220 nm^2^.

Figure 4 shows high resolution AFM topography images acquired at room temperature for the fresh sample without any thermal history and the sample after being thermally cycled eight times. The latter particles present significant surface damage (“peeling”) attributed to the repeated heating-cooling cycles and the related microstructural rearrangement. It is interesting to notice that the “surface peeling” allows us to perceive a sheet-like structure. The distance between these sheets was measured, obtaining heights of 1–2 nm. (Additional images of this fatigue phenomenon are presented in Figure S4 in the SM.) These images also give support to previous crystallographic studies, in which it was shown that even if their magnetic behavior remains stable, particles of **1** slowly lose their crystallinity after several cycles, which is referred to as structural fatigability [11,12].

## 3. Materials and Methods

For the synthesis of **1** an aqueous solution of Fe(BF_4_)_2_·6H_2_O (212 mg, 0.625 mmol in 0.5 mL H_2_O) was added dropwise to a mixture of 1.8 mL of Triton X-100, 1.8 mL of 1-hexanol and 4 mL of cyclohexane. An identical microemulsion was prepared with an aqueous solution of H-trz (131 mg, 1.875 mmol in 0.5 mL H_2_O). These two microemulsions were mixed together and left to stir for 24 h. The obtained nanoparticles were separated, washed three times with ethanol, sonicated for 30 min in ethanol (9 mg powder in 4 mL EtOH) and spin-coated over silicon substrates (speed: 4500 rpm, acceleration: 4000 rpm^2^, time: 30 s). Different coverages were obtained by spin-coating different amounts of particle suspensions. Magnetic susceptibility of the powder was measured using an MPMS (Quantum Design, San Diego, CA, USA) magnetometer in the 300–400 K temperature range under a magnetic field of 0.1 T with heating/cooling rates of ±1 K/min. The magnetic data were corrected to the diamagnetic contributions. Scanning electron microscopy (SEM) images were acquired at room temperature using a Hitachi S-4800 instrument (Krefeld, Germany). AFM images were acquired using a CYPHER-ES microscope (Oxford Instruments, Asylum Research, Santa Barbara, CA, USA) in amplitude-modulation mode in air between 293 and 393 K. Images were acquired either with OMCLAC160TS-R3 (Olympus, Tokyo, Japan, f = 300 kHz, k = 26 N/m, Al reflex coating) or ARROW-UHF-AuD (Nanoworld, Neuchâtel, Switzerland, f = 2 MHz, k = 6 N/m, Au reflex coating) probes, though the latter allowed for less invasive imaging conditions in most cases. The resonance frequency was re-calibrated at each temperature from the thermal noise of the cantilever. The amplitude set-point for approach was set at about 40% of the free amplitude.

## 4. Conclusions

We have succeeded in imaging the morphology changes associated with the phase transition in nanoparticles of the spin crossover complex [Fe(Htrz)_2_(trz)](BF_4_) using variable-temperature AFM in amplitude modulation mode. The relatively soft imaging conditions and accurate temperature control allowed us to acquire images over several thermal spin-state switching cycles without any significant tip-induced modification of the sample surface. Our measurements evidenced in a repeatable manner a very significant microstructural reorganization of the particles during the low spin to high spin transition, which involved a volume expansion, grain boundary changes and a certain degree of separation of the nanocrystallites, which were jointed together during the sample synthesis. These changes were found particularly important during the first switching event and only partially reversible during the reverse (HS to LS) switching. This result may thus explain the observed shift of the LS to HS transition temperature between the first and second heating cycles. In addition, we also observed a degradation of the particles manifested by the “peeling” of their surface layer as well as a slow recovery of the grain boundary morphology on a monthly scale. These findings highlight the importance of the investigation of microstructural changes in spin crossover compounds. Our results are in line with the fatigability of this compound inferred from previous powder X-ray diffraction studies. In this context, a word of caution is necessary as different samples with different particle size, shape, matrix, etc., may behave in different ways. Hence, for a better comparison it would be very useful to carry out simultaneous in situ magnetic, structural (XRD, Raman, etc.) and microstructural (AFM, SEM, TEM, etc.) investigations on the same sample across the same thermal treatments. Another interesting perspective is the quantitative investigation of the mechanical property changes by AFM, which seems to be particularly important in this sample as suggested by the previous XRD data.

## Figures and Tables

**Table 1:** Evolution of surface roughness parameters through the spin transition (3 × 3 μm^2^ area).

Spin State	LS (303 K)	HS (360 K)	LS (360 K)
Ra (nm)	28 ± 4	23 ± 6	28 ± 6
Rq (nm)	34 ± 5	29 ± 7	34 ± 7

## Supplementary Materials

The following are available online at www.mdpi.com/1996-1944/9/7/537/s1. Figure S1–S4: Supplementary AFM images.
