# Supplementary material for: In Situ AFM Imaging of Microstructural Changes Associated with The Spin Transition in [Fe(Htrz)2(Trz)](Bf4) Nanoparticles
Source: Materials (Basel). 2016 Jun 30;9(7):537. doi: 10.3390/ma9070537 (PMC5456918; doi:10.3390/ma9070537)
Supplement: Supplementary file 1 [file materials-09-00537-s001.pdf]

Supplementary Materials: *In-situ* AFM Imaging of Microstructural Changes Associated with the Spin Transition in $[Fe(Htrz)_2(Trz)](Bf_4)$ Nanoparticles

María D. Manrique-Juárez, Iurii Suleimanov, Edna M. Hernandez, Lionel Salmon, Gábor Molnár and Azzedine Bousseksou

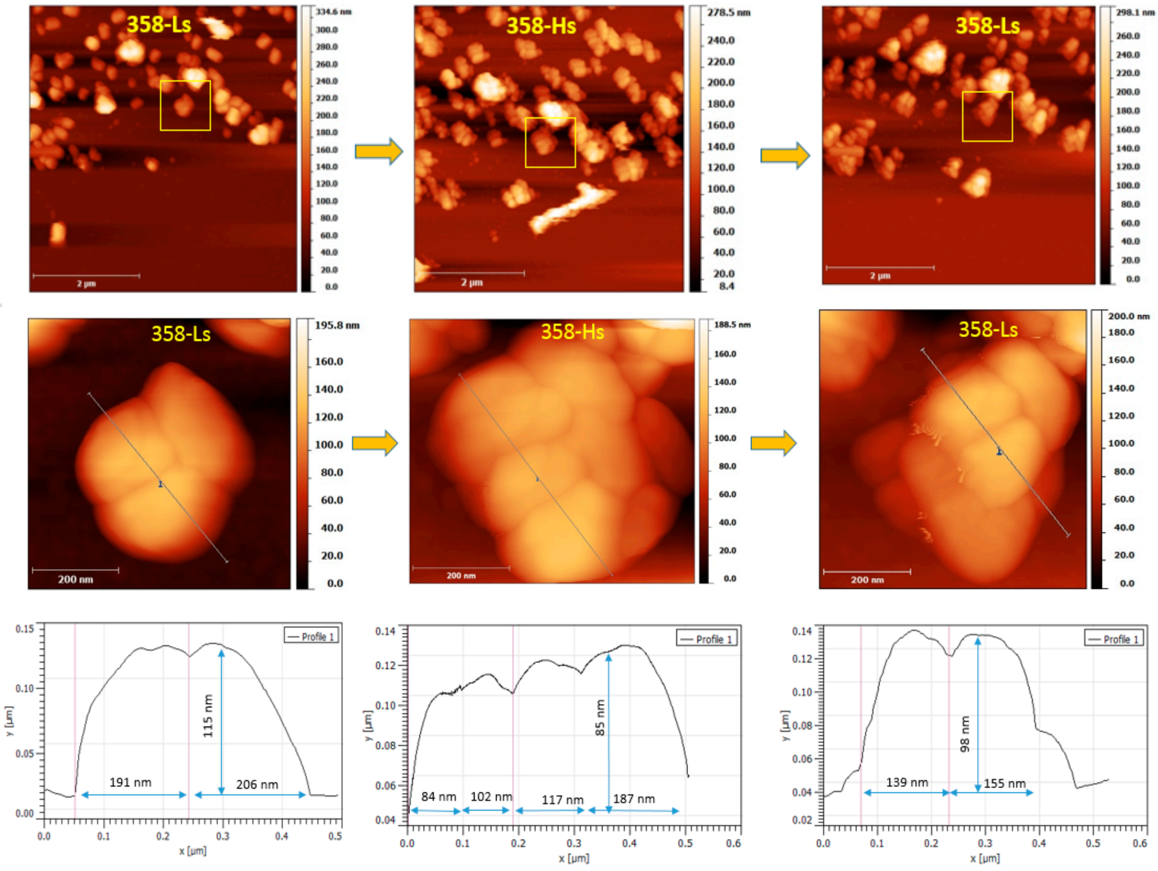

Figure S1. AFM height images and cross-sections of $[Fe(trz)(Htrz)_2](BF_4)$ nanoparticles acquired in different spin states at 358 K over a complete thermal cycle. (These data were extracted from the same experiment as Fig. 1 in the main text.) Images were acquired using a Cypher ES (Oxford Instruments) AFM and OMCLAC160TS-R3 probes (Olympus, f = 300 kHz, k = 26 N/m, Al reflex coating).

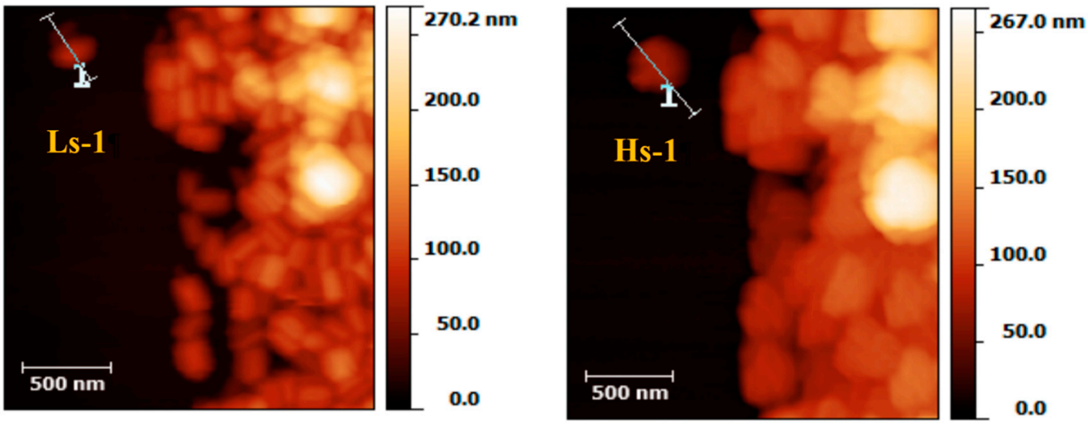

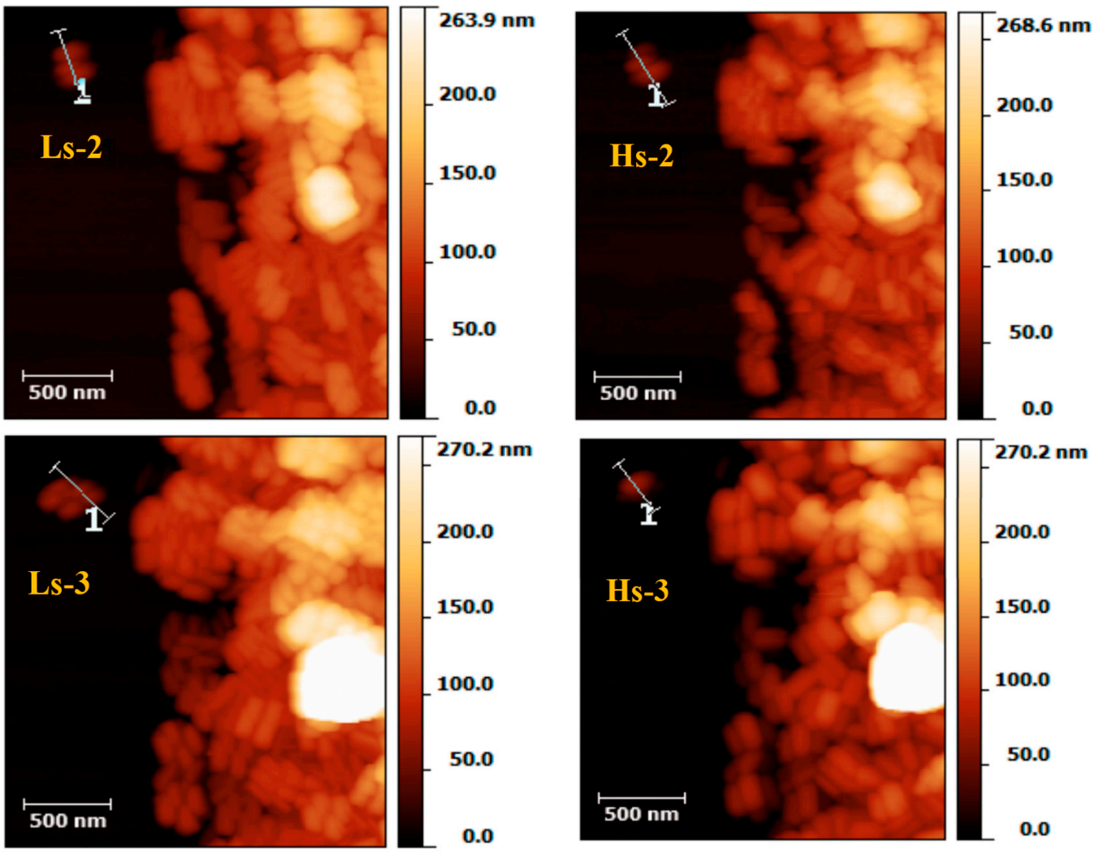

Figure S2. AFM height images of [Fe(trz)(Htrz)$_2$](BF$_4$) nanoparticles in the LS (left) and HS (right) states over three successive thermal cycles. Images were acquired using a Dimension Icon (Bruker) instrument and MPP-11120-10 probes (Bruker, f = 300 kHz, k = 40N/m, Al reflex coating).

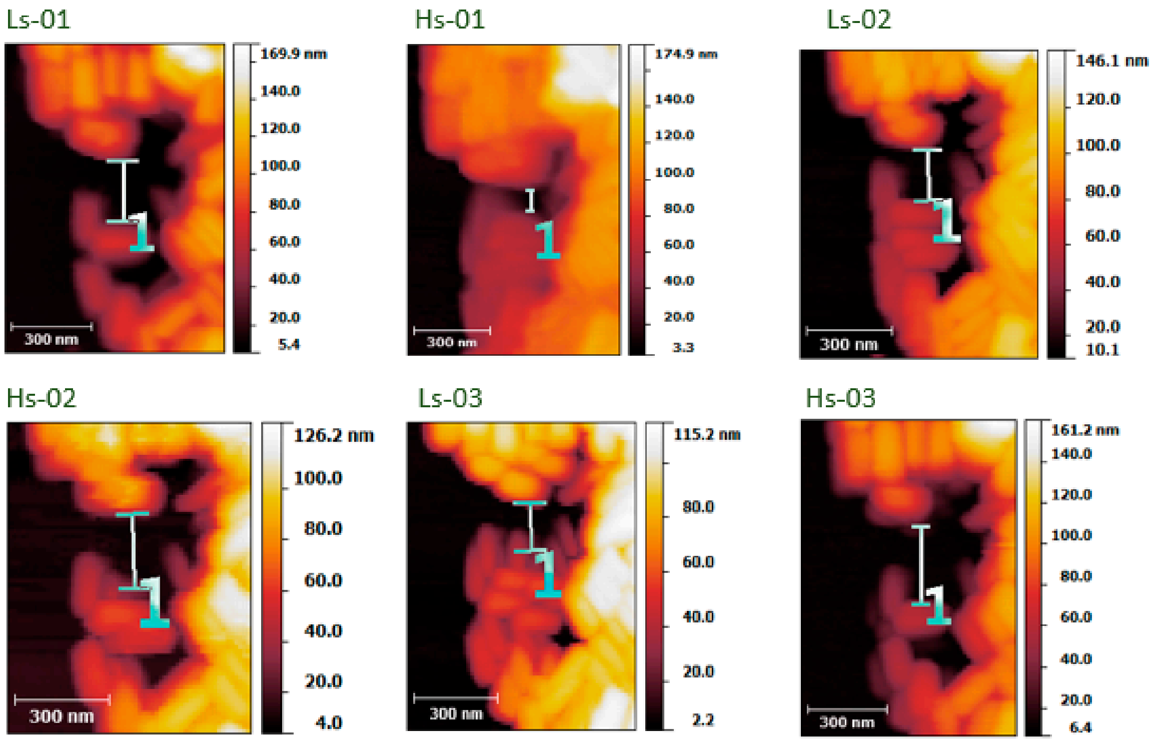

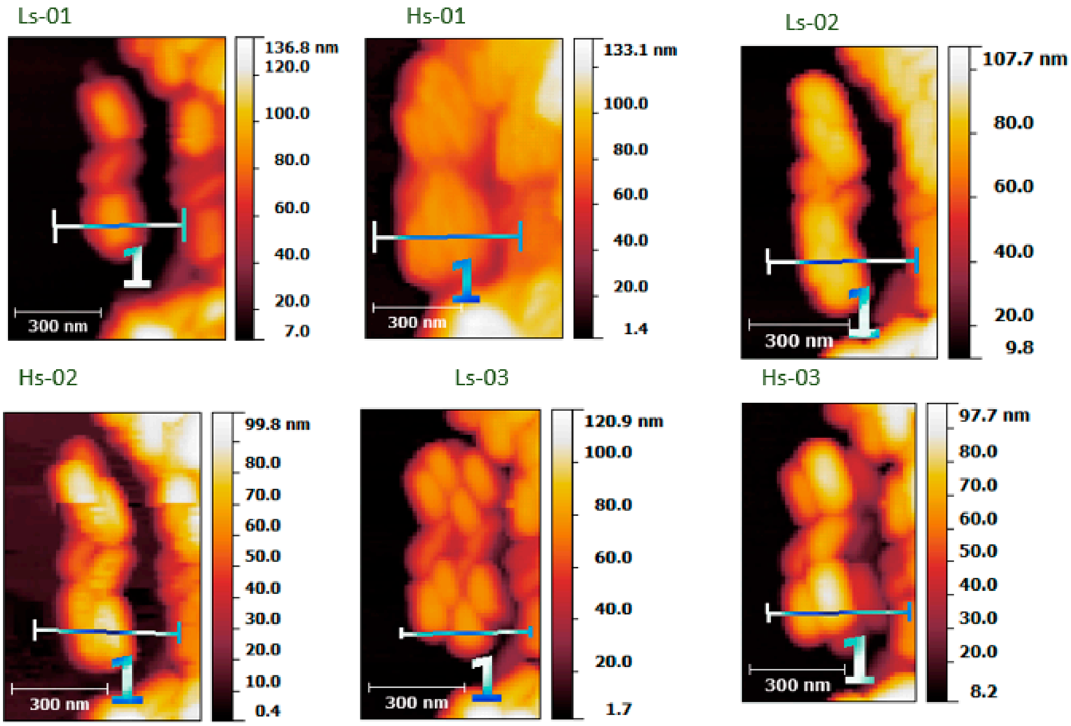

Figure S3. AFM height images of [Fe(trz)(Htrz)$_2$](BF$_4$) nanoparticles in the LS and HS states over three successive thermal cycles. (These data were extracted from the same experiment as Figure S2.)

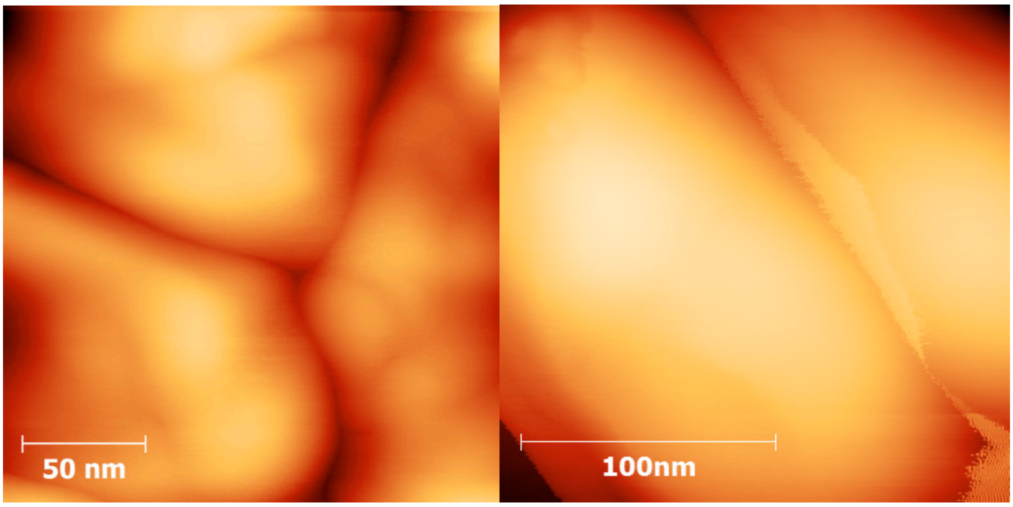

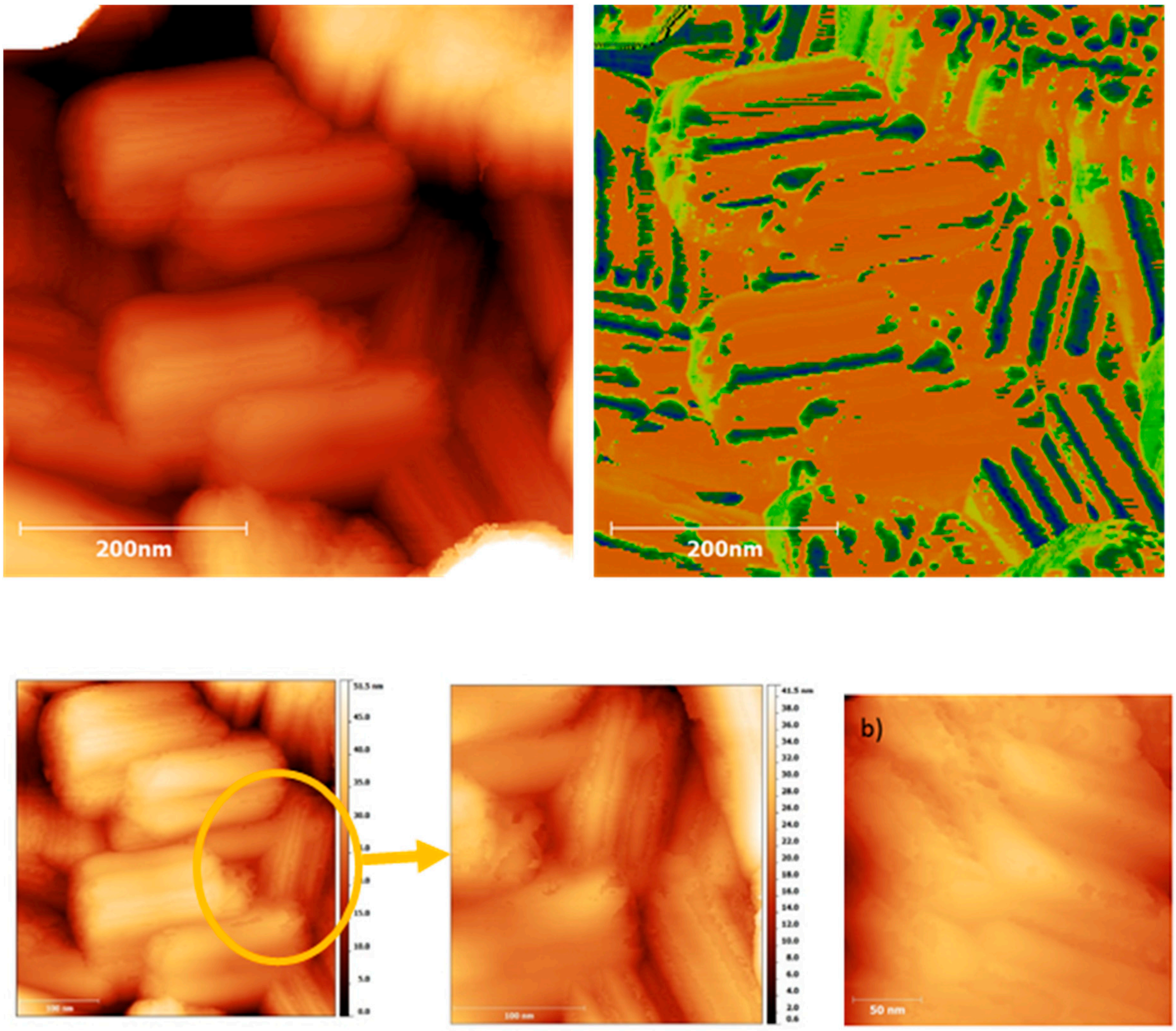

Figure S4. Surface degradation of [Fe(trz)(Htrz)$_2$](BF$_4$) particles following 8 thermal switching cycles: topography and phase images. The first two images were acquired on the fresh sample. Data were acquired using a Cypher ES (Oxford Instruments) AFM and ARROW-UHF-AuD (Nanoworld, f = 2 MHz, k = 6 N/m, Au reflex coating).

© 2016 by the authors. Submitted for possible open access publication under the terms and conditions of the Creative Commons Attribution (CC-BY) license (http://creativecommons.org/licenses/by/4.0/).
